# Meta-analysis is not always the best way to round out a systematic review: a few thoughts prompted by the COVID-19 pandemic and “spiced-up” with an earthquake

**DOI:** 10.3325/cmj.2020.61.198

**Published:** 2020-04

**Authors:** Vladimir Trkulja, Pero Hrabač

**Affiliations:** 1 Department of Pharmacology, Zagreb University School of Medicine, Zagreb, Croatia *vtrkulja@mef.hr*; 2 Department of Medical Statistics, Epidemiology, and Medical Informatics, “Andrija Štampar” School of Public Health, University of Zagreb School of Medicine, Zagreb, Croatia

The last few days of March 2020 in Zagreb, Croatia, were anything but usual early spring days. By the end of February, first patients with COVID-19 infection were identified, and hospitals were preparing for the (expected) increased number of patients, while most of them were considerably damaged by two strong earthquakes that hit Zagreb on March 22. Just about the end of March, a paper was published (1) that drew our attention – we considered that it might be useful to forward it to our hospital colleagues who did not have time to search for the literature that might guide their practice. A collaboration of several research groups resulted in a prompt, thorough, and up-to-date (at the time) systematic review focused on observational studies reporting on clinical, epidemiological, laboratory, and radiological characteristics and disease severity and course in COVID-19 patients (1). A thorough risk of bias assessment was performed using a tool adapted for this kind of studies. A total of 60 studies were finally included – 20 case reports, 37 case series, and 3 epidemiological reports involving between 1 and close to 50 000 patients per study, mostly from China, but also from 10 other countries (1). A number of meta-analytical estimates were generated in order to assess the prevalence of individual symptoms/signs, laboratory test values, and mortality – however, all were so severely heterogeneous that were completely non-informative. On the other hand, simply summarized data (as simple raw proportions), such as the percentage of patients with a certain laboratory value within or outside the physiological range, and narrative parts on certain findings were more informative (1).

Meta-analysis, as a research method, was broadly accepted in health care already in the 1980s (2), just a few years after its initial outline (3). There is no need to provide specific references for the following several statements, as they are self-evident: a) since then, an enormous number of meta-analytical methods have been developed (frequentist, Bayesian, direct, indirect, mixed-treatment, dose-response, for sparse data, meta-regression, etc); b) a number of user-friendly software packages have been developed; c) annual number of published meta-analyses pertaining to medicine is huge. The potential value of a systematic review (as a method of comprehensive identification and re-evaluation of primary research) and usually adjacent meta-analysis (as a method of data aggregation across different studies) in health care is undoubted and has been long recognized (eg 4). However, meta-analysis, as any other research method, is continuously evolving; initial “technical flaws” are being recognized and corrected, while new (potential) issues are continuously being recognized and solved. In other words, in order to do a proper meta-analysis, methodological expertise is required (although this may not seem so when one starts any of the “user friendly” software packages). More than 25 years ago one of the great contributors to the concept and methodology of meta-analysis noted as follows (2): “*There is currently a move to generate user-friendly meta-analysis software packages. I have mixed feelings about this development. On one hand, software is essential….(..). On the other hand, the ability to carry out multiple analyses routinely (note regression models for the case in point) often leads to poor data analysis. This, of course, should not be deterrent to creating good software. However, I would suggest a ‘Surgeon General’s warning’ on every software package: a meta-analysis is not to be undertaken lightly; it is not a simple procedure, nor is it a cure-all. It is time-consuming, requires specialists knowledgeable about the treatment working with statisticians, and is subject to abuse: treat it with respect*”. He in a way implied that meta-analysis could be methodologically (computationally) inadequate. Indeed, one (as an example of many) analysis (by expert methodologists) addressed several tens of thousands of meta-analyses in health care to conclude that around 20% of them were “flawed beyond repair” (5). However, the point that we would like to make is not strictly methodological one, rather a conceptual one – when should one actually do a meta-analysis, ie, after completing a systematic review how to decide whether data should be pooled or not? This does not refer to the issue of repeating research that has already been done [in the mentioned analysis (5) some 27% of published meta-analyses were judged as “redundant and unnecessary”] but to the issue of usefulness. Probably the most important purpose of a meta-analysis is to provide estimates that can improve the decision-making process, ie, to help define certain milestones that are relevant for practice (6). If this is not possible, if such an informative estimate cannot be generated – what is the use of a pooled estimate? [In the cited analysis (5) some 17% of meta-analytical estimates were considered “decent (presumably – technically), but not useful”]. Would it not be of more use to provide some other form of data synthesis? There are a number of situations in which this dilemma arises, and several are exemplified here. Assume a situation in which all primary studies are heavily flawed by high risks of various biases. What would be the purpose of a meta-analytical effect pooling? Aggregating “bias” simply cannot result in estimates that are likely to be close to the truth (7). Similarly, pooling estimates across highly biased and good quality trials is not really a reasonable option. Hence, data from individual unbiased studies are far more likely to be practically useful. Another common situation is one in which there are only a few primary studies. Since the fixed-effect estimates (underlying assumption: a single parameter is common to all primary studies) are rarely justified in biomedical sciences (8), a small number of studies represents a technical, computational problem – random-effects estimates would be needed and with just a few studies, estimation of the across-study variance (τ^2^) is highly imprecise, and this affects the overall estimate. Under such conditions, it may be more informative (and closer to the “truth”) to simply present individual study results (9), although there are methodological solutions that, however, require some expertise (10). This notion is directly linked to the typical situation of clinically (medically) heterogeneous primary studies (eg, different patient characteristics, study/treatment duration, outcome measurement instruments, and other differences in study features). They (regardless of “statistical heterogeneity or homogeneity”) conceptually require the use of random-effects estimation (8,10) (regardless of the number of studies). The assumption of one single population effect under so different circumstances is not realistic (8,10). Yet, while random-effects estimates do take into account between-study heterogeneity, they do not resolve it (8,10) – it comes to the proper understanding of such an estimate: the pooled random-effects estimate is an estimate of the mean of a range of population effects. When the number of primary studies is large, it is assumed that these effects are normally distributed, while when the number of studies is low, *t*-distribution is a more appropriate assumption (8,10). This fact further implies that some of these effects might not differ from 0 (or 1.0 for ratio effect measures) [ie, there is a range of true effects, some might be far from 0 (or 1), some could embrace it, some could go into the opposite direction]. Hence, unless the meta-analysis identifies settings (studies, subject characteristics, study characteristics, dosing, treatment duration, exact diagnosis, etc) in which there is an effect and those in which there is no effect, or settings with effects of different sizes – apart from the indication that, for example, some treatment “on average” produces an effect – the overall estimate may not be very informative for the practice since it does not “tell” under which circumstances the “treatment works,” and under which it “does not” or may be harmful. In such situations, the authors of meta-analysis should inform the readers about the dispersion of the estimated effects – by reporting the range of effects and prediction intervals – thus communicating the uncertainty (11,12).

To conclude, systematic reviews of the literature on a (bio)medical topic are important and potentially highly useful tools. The same goes for meta-analytical effect estimates. However, there are situations in which meta-analytical estimates are not really needed (ie, as a “final step” rounding out a systematic review) as they – due to the nature of the primary studies – may not be informative and relevant for the practice or, moreover, could be misleading. There are situations in which different ways of data synthesis (eg, narratives) or even data from individual studies (previously “filtered” on methodological grounds through the process of the systematic review) are much more informative and practically useful.
